# Intracellular molecular effects of insulin resistance in patients with metabolic syndrome

**DOI:** 10.1186/1475-2840-9-46

**Published:** 2010-09-01

**Authors:** Evasio Pasini, Vincenzo Flati, Silvia Paiardi, Damiano Rizzoni, Enzo Porteri, Roberto Aquilani, Deodato Assanelli, Giovanni Corsetti, Silvia Speca, Rita Rezzani, Carolina De Ciuceis, Enrico Agabiti-Rosei

**Affiliations:** 1 Salvatore Maugeri Foundation, IRCCS, Medical Center of Lumezzane, Brescia, Italy; 2 Clinica Medica, Department of Medical and Surgical Sciences, University of Brescia, Italy; 3 Metabolic Service and Nutritional Pathophysiology, S. Maugeri Foundation, IRCCS, Scientific Institute of Montescano (PV), Italy; 4 Department of Experimental Medicine, University of L'Aquila, Italy; 5 Section of Human Anatomy, Department of Biomedical Sciences and Biotechnology, University of Brescia, Italy; 6 Clinica Medica, Department of Medical and Surgical Sciences, University of Brescia c/o 2a Medicina Spedali Civili di Brescia Piazza Spedali Civili 1 25100 Brescia, Italy

## Abstract

**Aim of the study:**

Patients with metabolic syndrome (MetS) have an increased risk of cardiovascular disease. Data obtained from muscle biopsies have demonstrated altered insulin signaling (IS) in patients with MetS. The IS regulates critical cell functions including molecular-regulated cellular metabolite fluxes, protein and energetic metabolism, cell proliferation and apoptosis with consequent regulation of cell life including endothelial homeostasis and blood coagulation. However, little is known about blood cell IS in MetS patients. The aim of this study was to develop a method to evaluate IS in peripheral lymphocytes to identify altered intracellular molecules in patients with MetS to use as risk biomarkers of vascular thrombosis.

**Patients and Methods:**

We investigated 40 patients with MetS and 20 controls. MetS was defined according to guidelines from the US National Cholesterol Education Program Adult Treatment Panel III. Blood samples were taken from all participants. Total mononuclear cells were isolated from peripheral blood using density gradient centrifugation. IS molecules were evaluated using Western blot analysis followed by computer-assisted densitometer evaluation.

**Results:**

Lymphocytes of MetS patients showed a reduced mTOR expression (the mammalian target of rapamycin) which is a fundamental molecule of IS. Major impairment of IS was confirmed by reduced upstream and downstream mTOR molecules which regulate fundamental cells metabolic functions.

**Conclusions:**

In patients with MetS, we found a reduction of mTOR and other mTOR-related molecules involved in insulin resistance, cell repair, coagulation and vasculogenesis. A reduced expression of mTOR may reflect an increased risk of vascular thrombosis.

## Background

Metabolic syndrome (MetS) is a cluster of various clinical cardiovascular risk factors including obesity, dyslipidemia and hypertension, and is characterized by high fasting circulating insulin levels [1,2].

At present however, there are at least six operating definitions of MetS. Despite certain common features, these definitions differ for their relevant specific criteria [3]. Recently, concern has been raised for MetS, and no unifying cause of the syndrome has yet been identified [4]. Furthermore, the precise pathophysiological characteristics underlying the association of different cardiovascular risk factors in MetS remain elusive [5].

mTOR (the mammalian target of rapamycin) is a serine/tyrosine kinase. It is an important molecule of insulin signaling (IS) present in all cells. It lies at the center of the metabolic pathway and often operates in parallel to the cAMP pathway [6]

Indeed, IS is a very complex phenomenon which is influenced not only by the cellular metabolic status but also interacts with circulating molecules such as hormones (including insulin), nutrients and inflammatory molecules. mTOR and mTOR homologues are activated by both specific cytosolic signals which monitor cellular metabolic status and by extracellular circulating molecules. Accordingly, mTOR influences the energy metabolism, protein synthesis, cell cycles and reparative processes including anti-apoptotic effects which are fundamental for cell life span. Therefore, mTOR regulates the expression of adhesion molecules and pro-survival signals in both circulating and endothelial cells influencing blood circulation and clotting [7,8]. However, very little data are available on IS of circulating cells of MetS patients although clinical data show that inhibited mTOR, with specific inhibitors such as Serolimus or Everolimus after kidney transplantation, significantly increase the presence of *de novo *thrombotic micro-angiopathy with artery lesion characterized by intimal cell proliferation, necrosis and narrowed lumen. Complete withdrawal of mTOR inhibitors leads to improvement in many cases.

The increased incidence of vascular thrombosis when mTOR inhibitors are used and the improvement of micro-angiopathy when these drugs are withdrawn suggest the important role of mTOR in regulating vascular functions [8,9]

Recently, the effects of leptin on certain IS elements have been studied in human peripheral mononuclear cells in patients with MetS, given that the molecular mechanisms of IS are similar in all human tissue including lymphocytes [10]. In this study, the authors showed that leptin increases serine-138 phosphorylation of insulin receptor substrates-1 (IRS-1). Therefore, IS of insulin seems to be impaired at least at the IRS-1 level in MetS. However, the authors did not evaluate the downstream molecules involved in the intracellular insulin-mediated signaling including a fundamental molecule such as mTOR. This is particularly important because blood samples are easily collected, the procedure is repeatable and does not have the limitations of invasive approaches such as muscular biopsies.

Our aim was therefore to investigate the expression of relevant proteins involved in IS pathway such as mTOR. Since mTOR regulates blood/endothelial cells survival, vascular structure and function, and blood coagulation., it might be postulated its use as a risk biomarkers of thrombosis in MetS patients. In this study we applied, for the first time, a relatively simple and non-invasive method to evaluate IS in blood lymphocytes of patients with MetS.

## Patients and Methods

### Participants

Forty patients with MetS were recruited from subjects admitted to outpatient clinics. They underwent a comprehensive assessment of cardiovascular risk to establish the prevalence of risk factors. Patients with a previous diagnosis of diabetes mellitus or who were taking regularly lipid-lowering agents were excluded from the study.

The MetS and metabolic risks were defined according to the US National Cholesterol Education Program Adult Treatment Panel III guidelines [11] and modified as recommended in the latest American Heart Association/National Heart, Lung, and Blood Institute Scientific Statement [12] by adopting a lower cut-off for fasting glucose (≥5.6 mmol/L). The MetS was defined as having >3 of the following metabolic risk factors: 1) central obesity (waist circumference ≥88 cm in women and ≥102 cm in men), 2) hypertriglyceridemia (fasting triglycerides ≥1.7 mmol/L), 3) low HDL cholesterol (fasting HDL <1.3 mmol/L in women and <1.03 mmol/L in men), 4) glucose intolerance (fasting glucose ≥5.6 mmol/L), and 5) hypertension (sitting blood pressure ≥130/85 mm Hg obtained as a mean of 2 readings taken after resting for at least 10 minutes. Hypertensive patients were previously untreated. A group of 20 age and sex matched healthy patients were used as controls. Table 1 shows the baseline characteristics of the subjects. The study protocol was approved by the Ethics committee of our institution (University of Brescia Medical School), and informed consent was obtained from each participant, in accordance with institutional guidelines.

**Table 1:** Baseline characteristics of subjects according to the presence (MetS) or absence (controls) of metabolic syndrome.

	MetS (n = 40)	Controls (n = 20)	P
Sex (M/F)	32/8	14/6	NS
Age (years)	60 ± 3	58 ± 2	NS
BMI (Kg/m^2^)	30.1 ± 1.2	23.6 ± 0.8	P < 0.01
Blood pressure (mm Hg)	152/89 ± 2/1	129/79 ± 3/2	P < 0.01
Waist circumference	108 ± 5	82 ± 4	P < 0.01
Fasting glucose (mg/dl)	116 ± 7	87 ± 3	P < 0.01
OGTT (serum glucose after 120 min) (mg/dl)	187 ± 8	98 ± 4	P < 0.01
Serum cholesterol (mg/dl)	244 ± 5	186 ± 3	P < 0.01
Serum triglycerides (mg/dl)	207 ± 33	85 ± 20	P < 0.01
LDL cholesterol (mg/dl)	112 ± 7	100 ± 6	NS
HDL cholesterol (mg/dl)	47 ± 5	50 ± 3	NS
Serum creatinine	1.1 ± 0.1	0.9 ± 0.1	NS
Fasting insulin (μIU/mL)	12.0 ± 0.9	3.71 ± 0.4	P < 0.001
HOMA index	3.45 ± 2	0.8 ± 0.6	P < 0.01

OGTT: oral glucose tolerance test

HOMA index = Fasting insulin (μIU/mL)* fasting plasma glucose (FPG) (mmol/L)/22.5.

### Clinical and Biochemical Assessments

The subjects were examined after an overnight fast of at least 10 hours. Anthropometric measurements were made (height, weight, body mass index [BMI], waist circumference, and blood pressure) and biochemical variables were evaluated (fasting and 2-hour post-oral glucose tolerance test glucose, insulin, total cholesterol, triglycerides, and low-density lipoprotein - LDL and high-density lipoprotein - HDL cholesterol), as previously reported [13].

Insulin resistance was estimated with the homeostasis model assessment index (HOMA), calculated as fasting glucose (in mmol/L) times fasting insulin (in mIU/L) divided by 22.5. Erythrosedimentation speed was measured using standard methods.

### Isolation of human peripheral mononuclear cell

Human peripheral mononuclear cells (PMNGCs) were obtained by Ficoll-Paque density gradient centrifugation as described [10].

### Western Blots

Antibodies against mTOR was obtained from Sigma-Aldrich (Milano, Italy). Anti-p-p70S6K1, p-4E-BP1 and p-serine-636/639-IRS-1 were obtained from Cell Signaling Technology (Danvers, MA, USA). Anti-IRS-1 was obtained from Upstate (Charlottesville, VA, USA) and anti-Insulin Receptor alpha was from Santa Cruz Biotechnology (Heidelberg, Germany)

Total proteins were extracted from lymphomonocyte in lysis buffer (50 mM Tris.Cl pH 7.8, 1% Triton X100, 0.1% SDS, 250 mM NaCl, 5 mM EDTA, 100 mM NaF, 2 mM NaPPi, 2 mM Na_3_VO_4_, 1 mM PMSF). The crude lysate was centrifuged at 16000 g for 15 minutes, the supernatant was recovered and assayed for protein concentration by the Bradford Assay. Protein extracts were run on a 7.5% SDS-PAGE for IRS-1, p-serine 636/639-IRS-1, Insulin Receptor alpha and mTOR or 15% SDS-PAGE for p70S6K1, p-4E-BP1, p-p70S6K1 and transferred onto a PVDF membrane (Millipore, Milano, Italy). The membranes were stained with Ponceau Red (Sigma-Aldrich, Milano, Italy) and were blocked at RT for 2 hours with 10% non-fat dry-milk in TBST containing 0.1% Tween20. After this, the blots were washed briefly and incubated with primary antibodies directed either against p70S6K1 (1:500 for 2 hours at RT), IRS-1 (1:1500 O/N at 4°C), p-serine-636/639-IRS-1 (1:1500 O/N at 4°C), Insulin Receptor alpha (1:300 for 2 hours at RT), p-4E-BP1 (1:1500 O/N at 4°C), p-p70S6K1 (1:1000 O/N at 4°C), mTOR (1:3000 for 2 hours at RT) diluted with 5% non-fat milk or 5% BSA (only for the phosphor-specific antibodies) in TBST 0.1% Tween20.

The membranes were then washed 3 times for 10 minutes with TBST. Then, they were incubated for 1 hour at room temperature (RT), with anti-rabbit or anti-mouse (depending on the primary antibody) HRP-conjugated secondary antibody (Bio-Rad Laboratories, Milano, Italy) diluted 1/2000 in TBST containing 5% non-fat milk. The membranes were washed 3 times for 10 minutes, incubated in SuperSignal West Pico (Pierce Biotechnology, Rockford, IL, USA) chem-luminescent substrate and exposed to autoradiography films (Fuji Photo Film Co., Dusseldorf, Germany). The optical densities of blot bands were finally determined using a computer-assisted densitometer (Rasband, W.S., ImageJ, U. S. National Institutes of Health, Bethesda, Maryland, USA, http://rsb.info.nih.gov/ij/, 1997-2006).

### Statistical approach

Results are expressed as means ± SEM. The statistical analysis used one-way analysis of variance analysis. P-values less than 0.05 were considered significant. As it was a hypothesis-generating study no corrections for multiple comparisons were made. All variable were normally distributed.

## Results

The clinical characteristics of our population are summarized in Table 1. We selected subjects with many signs of MetS to identify intracellular molecular impairment responsible for their pathological conditions. Indeed, our MetS patients had higher blood pressure values, BMI, fasting glucose, triglycerides and total or LDL cholesterol levels as well as a greater prevalence of hypertension compared with controls. In addition, the HOMA index and the glucose levels after OGTT were higher in patients with MetS compared with controls suggesting that, as expected, insulin resistance was present in our subjects.

To avoid possible interference, of IS by other diseases, we excluded patients with renal or other significant problems. Indeed, serum creatinine and erythrosedimentation speed (data not shown) were normal in both groups.

**Figure 1:**
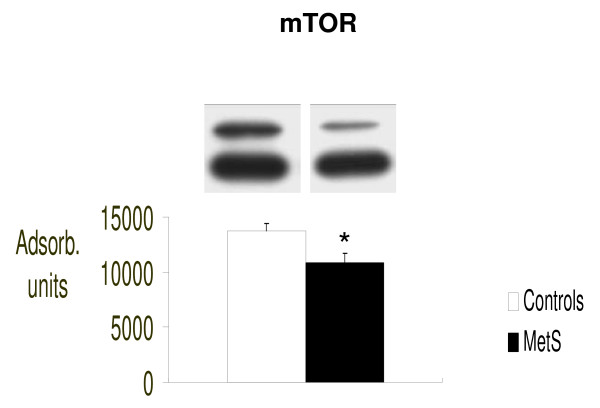
**Intracellular concentrations of mTOR evaluated by Western blot (absorbance units)**. *p < 0.05 vs. Controls.

**Figure 2:**
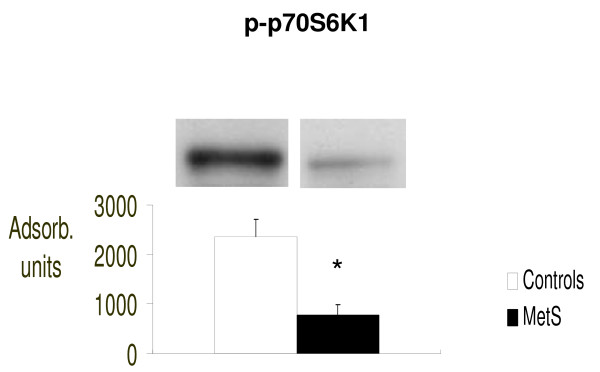
**Intracellular concentrations of phosphorylated-p70S6K1 (p-p70S6K1), evaluated by Western blot (absorbance units)**. *p < 0.05 vs. Controls.

**Figure 3:**
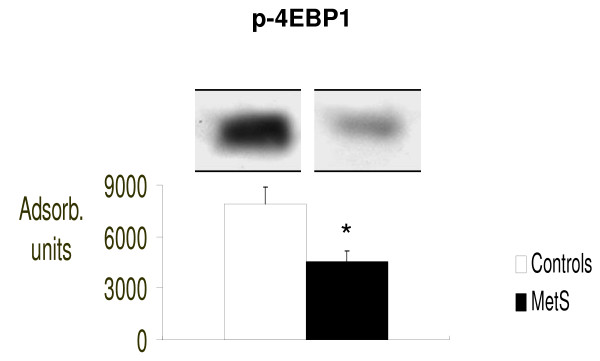
**Intracellular concentrations of phosphorylated-4E-BP1 (p-4E-BP1), evaluated by Western blot (absorbance units)**. *p < 0.05 vs. Controls.

We found that IS signaling was significantly impaired in our patients with MetS, as confirmed by significantly reduced molecular concentrations serine/tyrosine-kinase mTOR (p < 0.05 vs control) and its downstream effectors p70S6K1 and p-4E-BP1 (respectively: p < 0.02 and p < 0.05 vs controls). (Figure 1, 2 and 3 respectively).

**Figure 4:**
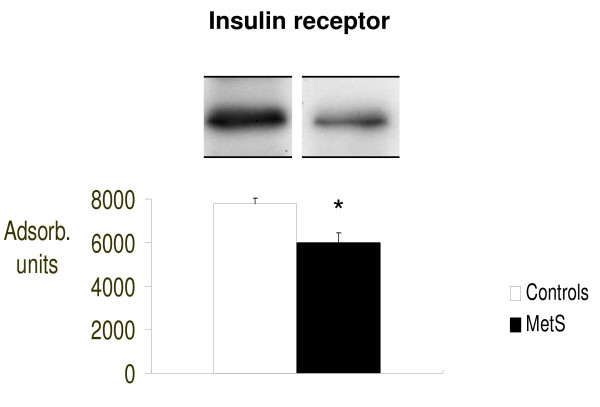
**Intracellular concentrations of Insulin Receptor, evaluated by Western blot (absorbance units)**. *p < 0.05 vs. Controls.

**Figure 5:**
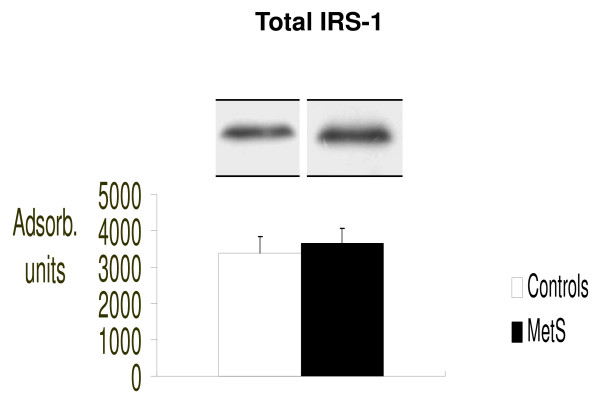
**Intracellular concentrations of total IRS-1, evaluated by Western blot (absorbance units)**.

**Figure 6:**
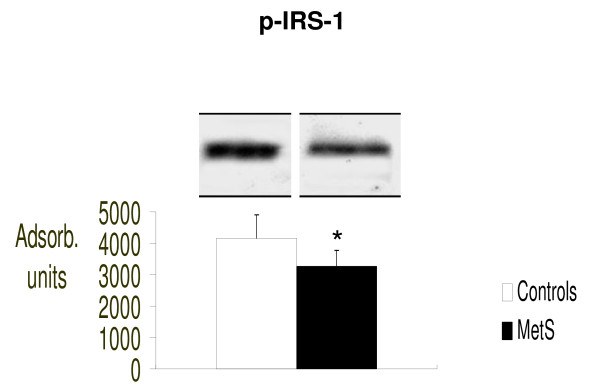
**Intracellular concentrations of serine-636/639-phosphorylated (inactive) form of IRS-1, evaluated by Western blot (absorbance units)**. *p < 0.05 vs. Controls.

In addition, we analyzed the molecules upstream of mTOR involved in cellular insulin signaling as illustrated in figure 4, 5 and 6. We therefore evaluated intracellular concentrations of insulin receptors, total IRS-1 and the serine-636/639 phosphorylated form of IRS-1 (p-serine-636/639-IRS-1). Figure 4 shows the insulin receptor expression, Figure 5 total IRS-1 and Figure 6 inactive form of IRS-1 in humans (serine-636/639-phosphorylated-IRS-1). We evaluated both total IRS-1 and its inactive form for a better idea of the insulin molecular cascade. We found that there were significantly fewer insulin receptors in patients with MetS (p < 0.03 vs. controls) suggesting a possible down-regulation process, as a consequence of the high blood insulin concentrations.

## Discussion

Our results strongly suggest that: a) it is possible to evaluate IS in blood cells such as lymphocytes, using a relatively simple and repeatable procedure, b) mTOR, which regulates replacement of damaged blood and endothelial cells with consequent maintenance of vasculature integrity and potential regulation of thrombotic phenomena and other molecules involved in the intracellular IS are significantly altered in patients with MetS. Consequently, mTOR cellular expression can be used to evaluate the disease status and the risk of vascular thrombosis.

### Intracellular Insulin Signaling

Reduced mTOR has many important consequences for cell metabolism and life span. Indeed, mTOR is stimulated by insulin signaling, nutrients, catabolic hormones, cytokines and growth factors [7]. It activates not only the protein synthesis through the phosphorylation of the enzyme p70S6K1 but also regulates important enzymes for cell life. Inhibition of mTOR reduces translocation of a subset of mRNAs and dramatically represses ribosomal mRNA and tRNA transcription. In addition, the use of specific mTOR blockers stop cell cycle progression in the early G1 phase of the cell cycle, driving cells into G0 state promoting apoptotic processes [14,15]. Moreover, mTOR not only avoids blood/endothelial cellular apoptosis but also probably repairs and replaces damaged endothelial cells stimulating endothelial progenitor cells with consequent maintenance of vascular functions including blood coagulation [8].

The role of mTOR in regulating blood coagulation has been recently clinically demonstrated. Indeed, cases of important *de novo *thrombotic micro-angiopathy have been reported in renal recipients treated with the mTOR inhibitor Sirolimus and Everolimus [8,9]. Treatment of micro-angiopathy is based on removal of these drugs. This would indicate that mTOR inhibition is crucial to decrease thrombotic events. Consequently, the significant reduction of mTOR found in MetS patients suggests that mTOR might be, in part, responsible for increased cardiovascular thrombotic diseases seen in MetS.

In addition, we know that mTOR and other molecules linked with this kinase, are influenced by circulating inflammatory cytokines and the nervous system. Indeed, cytokines such as TNF alpha - which are high in MetS-patient blood cause serine phosphorylation of IRS-1 and inhibit its tyrosine phosphorylation with consequent impairment of mTOR function [16].

Interestingly, Morisco *et al. *also recently demonstrated the presence of a cross-talk between β-adrenergic stimulation and IS by AKT, suggesting that there is an inter-relationship between the activation of the sympathetic nervous system and IS including AKT which influences mTOR function [17]. The role of mTOR and cross-talk with inflammatory and sympathetic systems and insulin signaling are very new and interesting observations and deserve further study to understand the molecular pathophysiology responsible for the increased cardiovascular disease associated with MetS. Moreover, we have recently shown that maintenance of cellular mTOR function by anti-hypertensive drugs improves insulin signaling increasing GLUT 4 expression and prevents micro-vascular rarefaction in spontaneously hypertensive rats with insulin resistance. This effect was independent of the reduction of blood pressure but was mTOR-related. [18].

We also found impairment of intracellular insulin signaling in patients with MetS. Indeed, insulin signaling is a complex phenomenon where mTOR plays a fundamental role. [5,7]. In detail, insulin binding to its specific receptor leads to the autophosphorylation of the trans-membrane β receptor sub-units and tyrosine phosphorylation of IRS-1 after their recruitment to the cell membrane. When IRS-1 is activated, it stimulates GLUT 4, with consequent regulation of glucose and lipid intracellular metabolism. In addition, activated IRS-1 modulates the phosphoinositide 3-kinase (PI3K) that in turn indirectly stimulates the activity of mTOR [19]. As discussed before, mTOR is a central regulator of cellular responses to hormones, growth factors and nutrients [7,20]. Current understanding of insulin signaling regulation considers IRS-1 to be a key protein in this cascade and mTOR activation.

The main cellular molecular mechanism of insulin desensitization, with consequent insulin resistance presents in MetS-patients, involves increased serine phosphorylation and decreased tyrosine phosphorylation of IRS-1. This is true in type 2 diabetic patients as well as in experimental models of insulin resistance. Phosphorylation of the tyrosine residues 608 on IRS-1 after insulin stimulation is necessary for propagation of the signal with consequent active-mTOR expression.

On the contrary, phosphorylation of serine residues leads to reduced insulin signaling [21,22]. It has therefore been proposed that changes in the equilibrium between serine or tyrosine phosphorylation lead to pathological conditions of insulin resistance and diabetes.

IRS-1 function is also negatively regulated by other circulating molecules found in the MetS such as catabolic hormones and inflammatory molecules [17]. Indeed, recent data has shown that the cytokine leptin promotes phosphorylation of serine 318 in IRS-1 in both skeletal muscle and in lymphocytes of obese and diabetic hyperleptinemic patients [10]. This would suggest: 1) that cytokines impair IRS-1 activity, blocking anabolic insulin signaling cascade with less activated mTOR and 2) that the molecular mechanism of leptin-mediated impairment of insulin signaling is similar in both skeletal muscle and lymphocytes.

Surprisingly, in our study p-serine-636/639-IRS-1 was significantly less in patients with MetS while there was a slight increased total IRS-1, although this was not statistical significant. We can explain these findings by considering that serine phosphorylated IRS-1 is rapidly eliminated in the cell cytoplasm like many other activated or deactivated molecules involved in intracellular signaling. Therefore, we probably observed the amount of p-serine-636/639-IRS-1 in the cell after its degradation. Indirect confirmation was the slightly increased total IRS-1, suggesting the cell's attempt to maintain adequate total concentrations of IRS-1 which could be activated or inactivated in response to specific stimuli. All these intracellular molecular data explain the phenomenon of insulin resistance present in patients with MetS.

Patients with MetS, in particular those with overt diabetes mellitus, are disproportionately affected by cardiovascular disease, compared with those without diabetes, due to a particularly pronounced atherosclerosis progression. Evidences suggest that insulin-resistance, diabetes and coronary heart disease share in common a deregulation of ubiquitin-proteasome system, a major pathway for nonlysosomal intracellular protein degradation in eucaryotic cells [23]. This might represent a common persistent pathogenic factor mediating the initial stage of the atherosclerosis as well as the progression to complicated plaque in diabetic patients [23].

In obesity and in diabetes mellitus, an increase in plasma free fatty acids, even still within the physiological range, might induces markers of endothelial activation, vascular inflammation and thrombosis [24]. Even transient and modest increases in plasma free fatty acids, also seen in healthy subjects may initiate early vascular abnormalities that promote atherosclerosis and cardiovascular disease [24]. Finally, also changes in the immune system might play a role in cardiovascular pathology. Biological processes altered in T cell aging are not only those typically associated with immune cells (T cell receptor signalling, cytokine-cytokine receptor interactions, etc.) but also some not specific of immune cells, such as peroxisome proliferator-activated receptors and mTOR signalling, as well as glucose and glutathione metabolism, suggesting that T cell aging may be representative of a more generalized aging phenomenon [25], with features characteristic also of the MetS.

Surprisingly enough, our patients with or without MetS do not differ significantly in the levels of HDL cholesterol, although patients with MetS tended to have lower values. We have no good explanation for this observation, apart from the relatively modest number of patients and subjects evaluated.

### The clinical perspective

MetS has many different clinical signs which include obesity, hypertension, diabetes and alteration of lipid metabolism [26]. Furthermore, recent research has shown that circulating molecules such as stress hormones and inflammatory cytokines increase in patients with MetS and they can influence and/or worsen IS including the central role of mTOR.

However, little is known about the intracellular molecular mechanisms present in MetS. We have demonstrated that IS is impaired in patients with MetS. Consequently, the observed molecular alterations can be used as biomarkers of this disease and its evolution.

We not only analyzed mTOR but also its downstream effectors p70S6K and 4EPB1 which stimulate anabolic pathway and other fundamental biochemical pathways such as the production of adhesion molecules, replace damaged cells and cell survival (including blood and endothelial cells with consequent regulation of blood coagulation). We also investigated the molecules which regulate important intracellular metabolic pathway such as cellular insulin stimulated molecules.

For mTOR evaluations we have developed a method that allows the study of IS in human peripheral mononuclear cells. We believe that our method has some relevant advantages; these are namely; 1) it is relatively easy to perform and may be repeated several time in the same subject, allowing the evaluation of time the time course of changes or the effect of treatment, 2) it avoids the pain or discomfort related to muscle biopsies, 3) it allows us to identify and quantify intracellular molecular damage and/or to study molecules which could link MetS, sympathetic activation and cell energy regulation. In addition, as it is repeatable, this method could be useful to assess the effects of interventions with specific therapeutic strategies such as drugs, weight reduction and/or physical training. Further investigation is needed to evaluate any correlations between intracellular molecular alterations and cardiovascular disease in a large scale study.

## Conclusion

In conclusion, using a relative simple and repeatable method, we analyzed intracellular molecules involved in IS and demonstrated impairments of important molecules as mTOR. mTOR modification is an important biomarker of cardiovascular risk factors not only because it compromises cell energetic metabolism and metabolic fluxes but also because mTOR regulates fundamental functions of blood and endothelial cell which, in turn, modulate blood/vascular interaction and integrity stimulating or avoiding vascular thrombosis.

## List of abbreviations used

MetS: Metabolic syndrome; IS: Insulin signalling; IRS-1: Insulin receptor substrates-1; LDL: Low-density lipoprotein; HDL: High-density lipoprotein; HOMA: Homeostasis model assessment index; (mTOR)mTOR: Mammalian target of rapamycin; cAMP: Cyclic Adenosin Monophosphate;

## Competing interests

The authors declare that they have no competing interests.

## Authors' contributions

all Authors have substantially contributed to the manuscript, either by handling data (EP, VF, SP, DR, EP, SS) or critically revising the manuscript (DR, RA, DA, CG, RR, CDC, EAR). All authors read and approved the final manuscript.
